# Non-invasive detection of *Orthohalarachne attenuata* (Banks, 1910) and *Orthohalarachne diminuata* (Doetschman, 1944) (Acari: Halarachnidae) in free-ranging synanthropic South American sea lions *Otaria flavescens* (Shaw, 1800)

**DOI:** 10.1016/j.ijppaw.2023.06.001

**Published:** 2023-06-03

**Authors:** Helena Rivera-Luna, Edwin Kniha, Pamela Muñoz, Javier Painean, Folko Balfanz, Stephan Hering-Hagenbeck, Heinrich Prosl, Julia Walochnik, Anja Taubert, Carlos Hermosilla, David Ebmer

**Affiliations:** a Institute of Parasitology, Biomedical Research Center Seltersberg, Justus Liebig University Giessen, Schubertstr. 81, 35392, Giessen, Germany; b Institute of Specific Prophylaxis and Tropical Medicine, Center for Pathophysiology, Infectiology and Immunology, Medical University of Vienna, Kinderspitalg. 15, 1090, Vienna, Austria; c Instituto de Patología Animal, Facultad de Ciencias Veterinarias, Universidad Austral de Chile, Valdivia, Chile; d Vienna Zoo, Maxingstr. 13b, 1130, Vienna, Austria; e Veterinary Clinic Vienna Zoo, Seckendorff-Gudent-Weg 6, 1130, Vienna, Austria

**Keywords:** Respiratory mites, Halarachnids, Pinnipeds, Molecular identification

## Abstract

Respiratory mites of the genera *Orthohalarachne* and *Halarachne* (Acari: Halarachnidae) are causative agents of nasopharyngeal/nasopulmonary acariasis in pinnipeds and sea otters. Until now, these endoparasitic mites were mainly diagnosed via necropsies and invasive procedures. So far, non-invasive diagnostic techniques have neither been developed nor applied in free-ranging pinnipeds. In the current study, we aimed to evaluate the halarachnid mite infestation status of free-ranging “urban” South American sea lions *Otaria flavescens* in the city of Valdivia, Chile. Therefore, non-invasive sampling methods were used in the current study, e. g. by observation-based sampling of freshly expectorated nasal mucus in the animal environment. Further, collection devices were developed for target-oriented sampling of sneezed nasal mucus, including sterile petri dishes and stretched clingfilms mounted on telescopic rods. Applying these techniques, 26 individual sputum samples were collected. 11.5% of sputum samples proved positive for halarachnid larvae (in total, n = 7), which were morphologically identified as *Orthohalarachne attenuata* (n = 2) or *Orthohalarachne diminuata* (n = 5). In one of the individual sea lion mucus samples, both *Orthohalarachne* species were detected, thereby confirming a patent co-infestation *in vivo*. 16S rDNA-based molecular identification of individual *Orthohalarachne* spp. larvae confirmed morphological findings. For the first time, we here molecularly characterized *Orthohalarachne* spp. on the basis of three gene regions [18S, 28S and the internal transcribed spacer 1 (ITS1)]. Overall, current data include the successful application of non-invasive techniques to sample halarachnid mites from free-ranging synanthropic pinnipeds and contribute to the current knowledge on respiratory mites infesting South American sea lions by combining morphological and molecular methods to overcome challenges in species identification. This study should further serve as baseline study and calls for more research on occurrence, biology and health implications of orthohalarachnosis in free-living as well as captive pinnipeds.

## Highlights

• Development of non-invasive methods to diagnose halarachnid mite infestations in free-ranging synanthropic pinnipeds.
• *In vivo* detection of an *Orthohalarachne* spp. co-infestation in a South American sea lion.
• First evidence of orthohalarachnosis in synanthropic South American sea lions in Valdivia, Chile.
• 16S-based molecular identification and first DNA sequence dataset (18S, 28S and ITS1) of *Orthohalarachne* spp.

## Graphical abstract

**Figure:**
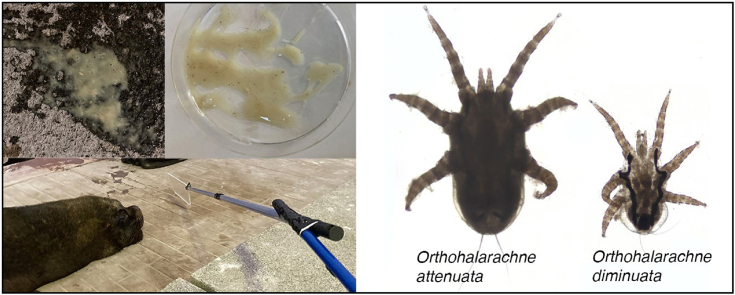

## 1 Introduction

Mesostigmatid mites of the genera *Orthohalarachne* Newell, 1947 and *Halarachne* Allman, 1847 (Acari: Halarachnidae) represent obligatory endoparasites and the causative agents of nasopharyngeal/nasopulmonary acariasis in pinnipeds and sea otters (Kim et al., 1980; Reckendorf et al., 2019; Rolbiecki et al., 2018; Seguel et al., 2018a, 2018b; Shockling Dent et al., 2019). Infestations can lead from asymptomatic to strong respiratory diseases accompanied by bacterial co-infections and eventual fatal outcomes (Dahme and Popp, 1963; Seguel et al., 2018a, 2018b; Ebmer et al., 2022). However, direct halarachnid mite-driven detrimental effects and their role as potential vectors and/or precursors of secondary bacterial, viral, and/or fungal infections are still not well known (Seguel et al., 2018a; Pesapane et al., 2022).

Transmission of halarachnid mites has been described to occur via different pathways, such as direct close contact (nose to nose) or transfer of expectorated mucus-infolded larval mites serving as motile transmission stages (Kim et al., 1980). Moreover, indirect transmission via zookeepers as mechanical vectors might play a role in zoological gardens and wildlife rescue centers (Pesapane et al., 2018). While mites of the genus *Halarachne* are specialized to parasitize phocids (earless seals) and sea otters, the genus *Orthohalarachne* parasitizes odobenids (walruses) and otariids (sea lions, fur seals) with two described species: *Orthohalarachne attenuata* (Banks, 1910) and *Orthohalarachne diminuata* (Doetschman, 1944) (Rolbiecki et al., 2018). Besides morphological differences in larval and adult mite stages (Furman, 1977; Kim et al., 1980), these two species also differ in their microhabitats (Kim et al., 1980). Hence, all stages of *Or. attenuata* mainly reside in the upper respiratory tract and infest nasal cavities and the nasopharyngeal mucosa, while *Or. diminuata* adults predominantly parasitize the lower respiratory tract (trachea, lungs), but their hexapod larvae and octopod nymphal stages (e.g. proto- and deutonymphs) inhabit the upper respiratory system (Kim et al., 1980). Co-infestations of both species have been recorded in different host species, including Northern fur seals *Callorhinus ursinus* (Linnaeus, 1758) (Kim et al., 1980), South American fur seals *Arctocephalus australis* (Zimmermann, 1783) (Gastal et al., 2016), and South American sea lions *Otaria flavescens* (Shaw, 1800) (Calderón-Mayo, 2015).

South American sea lions commonly occur in Atlantic (Argentina, Uruguay, southern Brazil and the Falkland islands) and Pacific (Chile, Peru) coastal regions (Vaz-Ferreira, 1982). In former centuries, these animals were intensively hunted and killed for train oil, leather and meat, leading to massive population declines especially in the early and mid 20th century (Grandi et al., 2015; Milano et al., 2020). Due to prohibition of sealing, *Ot. flavescens* populations started to recover (Grandi et al., 2015; Milano et al., 2020), being currently classified as “Least Concern” by the IUCN Red List (Cárdenas-Alayza et al., 2016). During the reproductive season, males lead harems encompassing up to 20 females or live in bachelor groups (Cárdenas-Alayza, 2018). In southern Chile, a unique bachelor group of synanthropic *Ot. flavescens* individuals has established in the city of Valdivia along the freshwater river Rio Calle-Calle, located approximately 15 km east of the Pacific coast. Currently, this “urban” colony is composed of around 70 individuals, living around the local fish market in close vicinity to humans. Previous studies on the parasite fauna of these animals demonstrated infections with potentially zoonotic endoparasite taxa (Hermosilla et al., 2016; Ebmer et al., 2020) and revealed the presence of the echinophthiriid louse *Antarctophthirus microchir* (Trouessart and Neumann, 1888) (Ebmer et al., 2019).

Up to date, the vast majority of studies on halarachnid mite infestations in free-ranging pinnipeds and sea otters are based on necropsies of stranded animals (Kim et al., 1980; Alonso-Farré et al., 2012; Gastal et al., 2016; Reckendorf et al., 2019; Shockling Dent et al., 2019; Pesapane et al., 2021). However, video-rhinoscopy was applied to diagnose halarachnid mite infestations in anesthetized otters (McDermott et al., 2013; Pesapane et al., 2022). In zoological gardens, respiratory mites have been reported as accidental findings during pathological dissections of pinnipeds (Kutzer and Burtscher, 1983; Ebmer et al., 2022). A first diagnostic approach showed the possibility to detect *Or. attenuata* infestations in trained captive walruses using video-endoscopy without the necessity of anesthesia (Fravel and Procter, 2016). However, video-based endoscopic techniques have only been used in captive/rehabilitated animals and no non- or minimally-invasive methods have been developed to sample halarachnid mites from free-ranging pinnipeds *in vivo*, so far.

In the current study, we aimed (*i*) to add new data on the occurrence of halarachnid mite infestations in South American sea lions and (*ii*) to develop and apply, for the first time, a non-invasive diagnostic method to sample respiratory mites in free-ranging synanthropic pinnipeds in a low-stress manner and in conformity with conservation and animal welfare aspects. By diagnosing orthohalarachnosis in both, free-ranging animals in Chile and a captive South American sea lion in the Vienna Zoo, Austria as recently published by our group (Ebmer et al., 2022), we here emphasize the importance of *in situ* and *ex situ* research projects.

## 2 Material & methods

### 2.1 Animal population

**Fig. 1:**
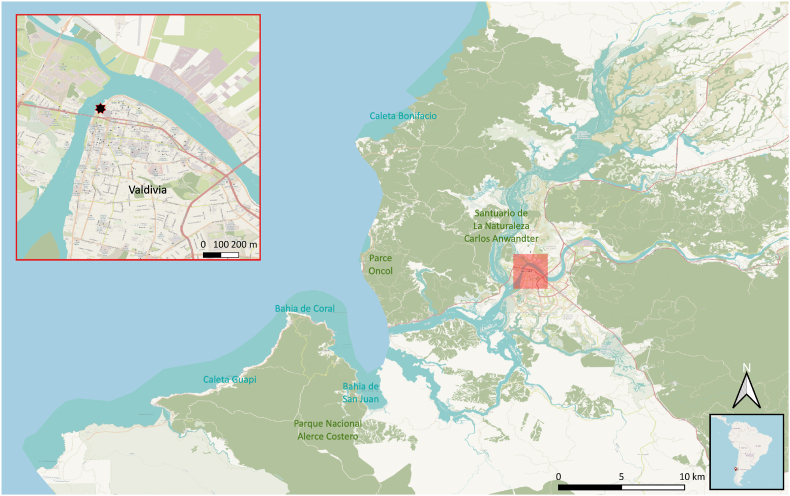
**Sampling area of *Orthohalarachne* spp. of South American sea lions in Valdivia, Chile.** The exact sampling location is shown in the section (upper-left) as a red-framed black star. Map created with QGIS (https://qgis.org/en/site/) and map data used from OpenStreetMap (openstreetmap.org/copyright). (For interpretation of the references to colour in this figure legend, the reader is referred to the Web version of this article.)

**Fig. 2:**
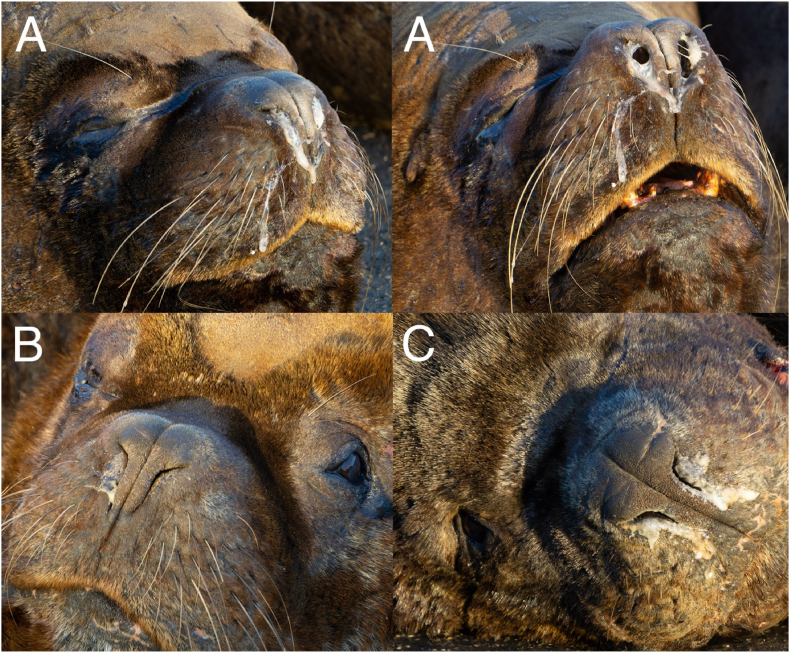
Nasal discharge in three individuals (A, B, C) of the “urban” colony of South American sea lions *Otaria flavescens* in Valdivia, Chile.

During recent non-invasive studies on the endo- and ectoparasite fauna of synanthropic South American sea lions in Valdivia, Chile (Fig. 1) (Hermosilla et al., 2016; Ebmer et al., 2019, 2020), severe respiratory symptoms were noticed in various animals of this group, including extensive mucous nasal discharge (Fig. 2) and marked sneezing and coughing episodes with clearly visible expectorations. These expectorations were also observed in the environment between feces and urine of the animals. Based on these longstanding observations, the current respiratory mite infestation status of the “urban” sea lion colony was subject of the present study. Therefore, favourite resting places of these sea lions at the waterside promenade along the freshwater river Rio Calle-Calle within the city center of Valdivia, Chile (“Nueva constanera”; 39°48′34.704″S, 73°14′46.247″W; Fig. 1) were chosen as main observing points and sampling spots. *Otaria flavescens* males have been known to colonize the city of Valdivia for more than 40 years by now (Schlatter, 1976). They represent a tourist attraction around the local fish market, where they are fed by the marketeers (Hermosilla et al., 2016). The behavior of these animals is well adapted to the urban lifestyle (e. g. climbing stairs out of the water, resting at public places) and they regularly interact with other domestic animal and wildlife species (e. g. stray dogs, water birds, rodents) and the local human population. Despite their synanthropic lifestyle, these sea lions are still free-ranging animals (Hermosilla et al., 2016).

### 2.2 Development of non-invasive techniques for the detection of halarachnid mites

**Fig. 3:**
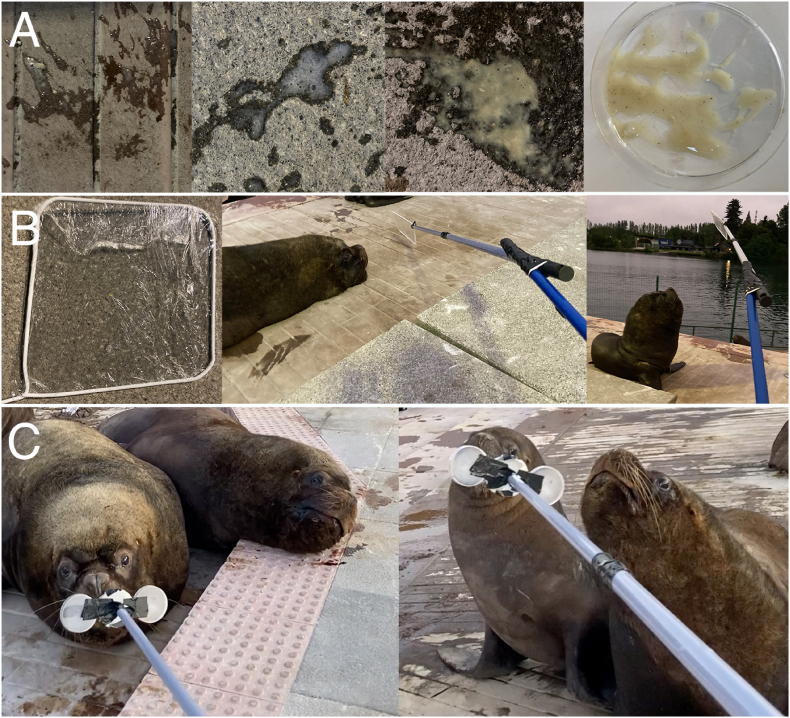
**Non-invasive diagnostic techniques for the detection of *Orthohalarachne* spp.** (A) Sampling of sneezed mucus droplets and mucous nasal discharges from substrates of resting places. (B) Metal clothes hanger bent to form a square frame, covered with clingfilm and mounted on a telescopic rod and (C) sterile petri dishes mounted on a telescopic rod to directly collect sputum samples from the animals.

Due to the current lack of non-invasive diagnostic methods of halarachnid mite infestations in free-ranging pinnipeds, we developed and applied different novel sampling approaches in the current study: (*i*) extensive animal behavioral observations served as basis for the sampling of freshly expectorated nasal mucus in the animal environment (Fig. 3A); (*ii*) using self-constructed collection devices for direct sampling of nasal expectorations consisting of clingfilm stretched on a clothes hanger bent to form a square frame (Fig. 3B) or conventional sterile petri dishes (Fig. 3C), both mounted on telescopic rods.

For current sample collection, members of this “urban” sea lion group were observed on a daily basis between September and October 2022. Sampling took place early in the morning, approximately 30 min before and after sunrise. These time points were recognized as ideal sampling times, since the cleaning of public places, tourists and traffic during the day were identified as confounding factors for the animals. In general, especially sleeping and resting animals were observed and their individual characteristics and respiratory tract-associated symptoms, like nasal and ocular discharge, sneezing or coughing were noted. Thus, exact descriptions helped to identify single animals for successful individual mucus sample collection. Additionally, attention focused on animals that were easily accessible, calm and did not show aggressive behavior. The animals were never fed or lured for sampling procedures.

During animal observation, it was important to recognize the beginning of intensive sneezing periods that started with marked and characteristic movements of the thoracic region during one or multiple strong inspiration phases. In this phase, uprighting movements were regularly recorded and resting animals changed their position from lying to sitting. To estimate the direction, range and amount of nasal mucus that was hence expectorated within subsequent expiration periods, exact observation by and agile movements of researchers proved essential to locate nasal expectorations in the environment. Thereby, it was often observed that phlegm was ejected over a distance of several meters before landing in the surrounding environment and/or on other sea lion individuals. Expectorated samples were then collected from the substrate after the sea lions had left the sampling sites (Fig. 3A) using spatula, dissection needles and pipettes and were stored in petri dishes for subsequent stereomicroscopic-based screenings for halarachnid mites.

For direct mucus sampling, different collection devices (see above) were mounted on telescopic rods to ensure a safe distance between animal and investigator and offer animals the possibility to leave the sampling spot at any time (Fig. 3B and C). In order to gain trust, the collection devices were several times presented to the animals, giving them the chance to explore the tool. When animals started sneezing, the device with attached collection surfaces was held approximately 30 cm in front of the sea lions’ head to directly collect nasal expectorations. Mucus samples were stored in the clingfilm by detaching the film from the clothes hangers and folding it for transportation. For the samples collected in petri dishes mounted on the telescopic rod, the dishes were detached and closed to store the samples for further analyses.

### 2.3 Analyses of mucus samples and morphological mite identifications

Mucus samples were screened for the presence of respiratory mites using both pocket loupes in the field and a stereomicroscope in the lab. Detected respiratory mites were preserved in 80% ethanol. Specimens were documented and morphologically identified based on morphological characteristics (Doetschman, 1944; Popp, 1961; Domrow, 1974; Furman, 1977) using a light microscope (Olympus BX50; Olympus, Tokio, Japan) equipped with a digital camera (Olympus EP50; Olympus, Tokio, Japan). After morphological identification, individual mites were stored in separate tubes for further molecular examinations.

### 2.4 Molecular identification and characterization

For DNA isolation, individual specimens were longitudinally cut with sterile needles and incubated in 180 μL ATL Buffer and 20 μL Proteinase K at 56 °C for 4 h. Subsequently, a DNeasy Blood & Tissue Kit (Qiagen, Hilden, Germany) was used following the manufacturer's instructions with a final elution volume of 100 μL. The quality of the isolated DNA and the quantification of the DNA concentration were assessed by using a NanoDrop Spectrophotometer ND-1000 (former PeqLab Biotechnologies, Erlangen, Germany).

Molecular identification was based on amplification of a 16S rRNA gene segment using the primer combination 16S + 1_orthohala (Ebmer et al., 2022) and 16S-1 (Black and Piesman, 1994). For samples with low DNA yields, the primer combinations 16S + 1_orthohala/16S-2 and 16S + 2/16S-1 were used to amplify shorter overlapping fragments as stated by Black and Piesman (1994) using the same PCR conditions (Table 1). To promote availability of currently insufficient molecular data of *Orthohalarachne* species, we additionally amplified multiple gene regions, 18S rDNA, 28S rDNA, internal transcribed spacer 1 (ITS1), by using published primers and modified PCR protocols (summarized in Table 1).

**Table 1:** PCR protocols for molecular analysis of *Orthohalarachne* species.

Locus	Primer sequence (5′-3′)	Amplicon size (bp)	Amplification protocol	Reference
16S	16S + 1_orthohala: CTGCTCAATGATTTTTTAAATTGCTGTGG 16S-1: GCTGTGGGATCATTTACCG	∼460 bp	94 °C 5 min; 38x: 94 °C 1 min, 52 °C 1 min, 72 °C 1 min; 72 °C 10 min	Black and Piesman (1994); Ebmer et al. (2022)
	16S+2^a^: TTGGGCAAGAAGACCCTATGAA 16S-2^a^: TTACGCTGTTATCCCTAGAG			
18S	31F1: CGCGAATGGCTCATTAAATC 344R2: GCCTTCCTTGGATGTGGTAG	∼290 bp	95 °C 15 min; 35x: 95 °C 30 s, 53 °C 30 s, 72 °C 1 min; 72 °C 10 min	Foley et al. (2013)
28S	43F: GCTGCGAGTGAACTGGAATCAAGCCT 929R: AGGTCACCATCTTTCGGGTC	∼860 bp	95 °C 15 min; 35x: 95 °C 30 s, 53 °C 30 s, 72 °C 1 min; 72 °C 10 min	Dowling and OConnor (2010)
ITS1	ITS1_F: AGAGGAAGTAAAAGTCGTAACAAG ITS1_R: ATATGCTTAAATTCAGGGGG	∼470 bp	95 °C 15 min; 35x: 95 °C 30 s, 53 °C 30 s, 72 °C 1 min; 72 °C 10 min	Morelli and Spicer (2007)

a Primer combination 16S + 1_orthohala/16S-2 and 16S + 2/16S-1 result in ∼300 bp including primer.

PCR amplifications of the 16S rRNA gene were performed using a 2x EmeraldAmp® GT PCR Master Mix (Takara Bio Europa SAS, Saint-Germain-en-Laye, France) with 3–5 μL template DNA and sterile H_2_O adding up to a final volume of 25 μL. Moreover, fragments of the 18S rRNA gene, the 28S rRNA gene and the internal transcribed spacer 1 region were amplified using 10 x Reaction Buffer B with 2.5 mM MgCl_2_, 1.6 mM dNTPs, 1 μM primers, 1.25 units DNA polymerase and 3–5 μL DNA, adding sterile H_2_O to a final volume of 50 μL.

All PCR amplifications were run on an Eppendorf Mastercycler (Eppendorf AG, Hamburg, Germany). Bands were analyzed with a Gel DocTM XR + Imager (Bio-Rad Laboratories, Inc., California, U.S.A.), cut out, purified with an IllustraTM GFXTM PCR DNA and Gel Purification Kit (GE Healthcare, Buckinghamshire, UK), and Sanger sequenced by using a BigDye® Terminator v.1.1 Cycle Sequencing kit (Thermo Fisher Scientific Inc, Waltham, MA, USA) and run on a SeqStudio® Genetic Analyzer (Thermo Fisher Scientific Inc., Waltham, MA, USA).

Sequences were obtained from both strands, aligned with Clustal X 2.1 (Larkin et al., 2007), and a consensus sequence was generated in GenDoc 2.7.0 (Nicholas, 1997). The obtained sequences were uploaded to GenBank (Table 2) and compared to available sequences in the GenBank database using the Basic Local Alignment Search Tool (BLAST) (https://blast.ncbi.nlm.nih.gov/Blast.cgi). Available 16S rDNA reference sequences were downloaded from GenBank for comparison with obtained sequences and aligned using ClustalX 2.1 for multiple alignment and GeneDoc 2.7.0 for manual editing. To identify unique haplotypes among species, DnaSP v.5 (Librado and Rozas, 2009) was used and respective TCS haplotype networks were generated with Popart (Leigh and Bryant, 2015). Uncorrected pairwise distances were calculated with MEGAX (Kumar et al., 2018). To confirm species delimitation based on 16S rDNA, obtained sequences were compared with reference sequences by using the Automatic Barcode Gap Discovery (ABGD) web-interface program (https://bioinfo.mnhn.fr/abi/public/abgd/), which generates Kimura-2-parameter (K2P) distances and assigns sequences to hypothetical species. Default settings of intraspecific divergence (P) of 0.001–0.1 were applied (Puillandre et al., 2012).

**Table 2:** Molecular characteristics of analyzed *Or. attenuata* (n = 2) and *Or. diminuata* (n = 5) sequences.

Species	Locus	Amplicon size (bp)^a^	Haplo-types	Accession numbers	Blast results (% identity)^b^
*Or. attenuata*	16S	383	1	OQ376656, OQ376657	*Or. attenuata* (96.35%–96.88%)
	18S	285	1	OQ423275, OQ423276	*Chiroptonyssus* spp. (98%), *Ornithonyssus* spp. (97%), *H. halichoeri* (96%)
	28S	840	1	OQ435875, OQ435876	*Gaeolaelaps* spp. (93%) *H. halichoeri* (92%) *Hypoaspis* spp. (91%)
	ITS1	512	1	OQ435881, OQ435882	*Coleolaelaps* spp. (92%) *H. halichoeri* (86%)
*Or. diminuata*	16S	383	1	OQ376658–OQ376661, OQ592162	*Or. diminuata* (100%)
	18S	285	1	OQ423277–OQ423280, OQ586386	*Aetholaelaps* spp. (97%) *Macrocheles* spp. (97%) *H. halichoeri* (96%)
	28S	840	1	OQ435877–OQ435880	*Gaeolaelaps* spp. (94%) *H. halichoeri* (93%) *Hypoaspis* spp. (92%)
	ITS1	488	3	OQ435883–OQ435886	*Coleolaelaps* spp. (92–93%) *H. halichoeri* (90–91%)

a without primers.

b top hits (E value) including Halarachnidae sequences, if available.

## 3 Results

### 3.1 Non-invasive sampling and halarachnid mite identification

Overall, 26 individual expectorated nasal mucus samples were collected. These included 21 samples collected in the environment after intensive animal observation and five samples directly collected via the newly developed collection devices. In total, 11.5% (3/26) of individual sea lion samples proved positive for respiratory mites. Hence, one, two and four larvae were found in three positive environmental samples.

**Fig. 4:**
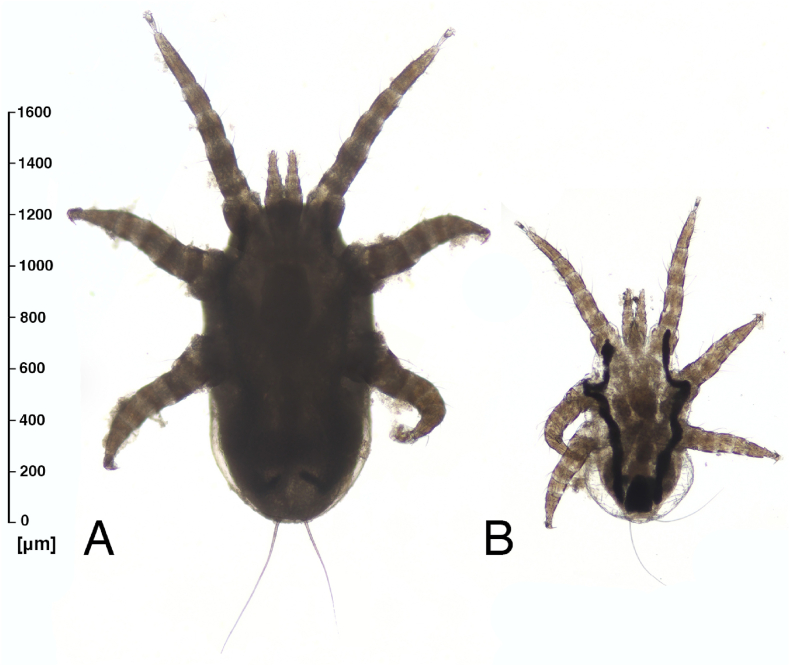
Larval stages of (A) *Orthohalarachne attenuata* and (B) *Orthohalarachne diminuata* showing distinct differences in idiosoma length.

The morphological identification of arthropods detected in nasal expectorations revealed the presence of seven larval stages of *Orthohalarachne* spp., including two *Orthohalarachne attenuata* and five *Orthohalarachne diminuata* specimens. In one of three individual sea lion nasal mucus samples, a co-infestation was diagnosed since it contained two *Or. diminuata* and two *Or. attenuata* larval stages (Fig. 4). The other two positive nasal expectorations contained one and two *Or. diminuata* larval stages, respectively. Microscopic measurements revealed an idiosoma length of 948–1228 μm for *Or. attenuata* and 661–905 μm for *Or. diminuata* larvae, while gnathosoma length was 312–317 μm in *Or. attenuata* and 193–261 μm in *Or. diminuata* larvae.

### 3.2 Molecular identification and characterization

All seven samples could be successfully amplified by all applied PCR protocols and full length sequences were obtained. Sample OC5 showed low DNA yields (∼1 ng/μL vs. ∼5 ng/μL for others) and the full 16S sequence were obtained from overlapping fragments. Based on the obtained 16S rDNA sequences, two specimens were molecularly identified as *Or. attenuata* (OC2 and OC3) and five specimens as *Or. diminuata* (OC1, OC4–OC7). For *Or. attenuata*, sequence identity ranged from 96.35 to 96.88% compared to available sequences in GenBank (MZ435794.1 and MZ435798.1, respectively), whereas for *Or. diminuata,* 100% identity was observed with reference sequences (OP087659.1, OP087662.1) (Table 2).

Amplicons and Blast results of the 18S, 28S and ITS1 gene regions are summarized in Table 2. Sequenced *Orthohalarachne* mites showed 86–98% identity with other mesostigmatid mite genera of the superfamily Dermanyssoidea. Sequence identities of both analyzed *Orthohalarachne* species with available *Halarachne halichoeri* sequences were 96% for 18S, 92–93% for 28S, and 86–91% for ITS1 (Table 2).

**Fig. 5:**
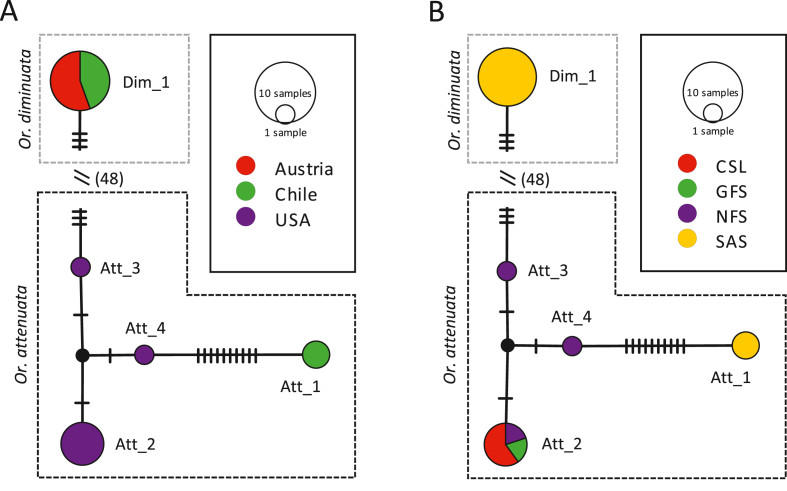
**Haplotype (TCS) networks of *Orthohalarachne diminuata* and *Orthohalarachne attenuata* based on 16S rDNA sequences.** (A) Network analysis based on countries of origin, (B) network analysis based on the pinniped host species (CSL=California sea lion, GFS = Guadalupe fur seal, NFS=Northern fur seal, SAS=South American sea lion). For better visualization a combined network analysis of *Or. attenuata* and *Or. diminuata* sequences is shown, however, the calculated distance (48 mutations) between species was clipped. *Or. attenuata* haplotypes are encircled in black boxes with dashed lines, whereas *Or. diminuata* haplotypes are encircled in light grey boxes with dashed lines based on estimated MOTUs by ABGD.

For further molecular comparison of *Or. attenuata* and *Or. diminuata* originating from different countries, reference 16S rDNA sequences were obtained from GenBank. Altogether, 18 sequences were included in the analysis (seven additional *Or. attenuata* and four additional *Or. diminuata*), aligned and trimmed to a total length of 369 bp. For *Or. attenuata*, four haplotypes showing 13 segregating sites were observed with an overall haplotype diversity (Hd) of 0.694 and a nucleotide diversity (π) of 0.018. Both *Or. attenuata* sequences obtained from Chilean specimens formed a unique haplotype (Att_1). While haplotype Att_2 comprised five sequences, haplotype Att_3 and Att_4 comprised only one sequence each of *Or. attenuata* specimens obtained from pinniped populations in California, USA (Fig. 5A). Comparing haplotype Att_1 with all other *Or. attenuata* haplotypes, a deletion at alignment position 214 was observed, while haplotypes Att_2, Att_3, and Att_4 consisted of thymine (T) at alignment position 214 (Additional file: Fig. S1). For *Or. diminuata*, only a single haplotype (Dim_1) was observed comprising sequences of specimens from Chile and Austria (Fig. 5A). Using an intraspecific threshold value of 0.05 (P), ABGD partitioned all sequences into two species.

*Or. attenuata* sequences originated from specimens parasitizing four different pinniped species. Att_1 sequences were obtained from *Or. attenuata* infesting South American sea lions [(SAS); *Otaria flavescens*], whereas Att_2 sequences originated from specimens infesting multiple pinniped species, namely California sea lions [(CSL); *Zalophus californianus*], Guadalupe fur seals [(GFS); *Arctocephalus townsendi*], and Northern fur seals [(NFS); *Callorhinus ursinus*]. Att_3 and Att_4 solely originated from NFS hosts (Fig. 5B). All *Or. diminuata* sequences (Chilean and Austrian) originated from specimens infesting South American sea lions (Fig. 5B).

The overall mean genetic distance between all included *Or. attenuata* sequences was 1.4% (S.E. = 0.4%). Uncorrected intraspecific pairwise distances ranged from 0.0% to 3.3%. While pairwise distances between *Or. attenuata* specimens from pinniped populations in Chile and the USA ranged from 2.7% to 3.3%, genetic distances among specimens solely originating from populations in the USA ranged from 0.0% to 0.5% (Additional file: Table S1). As all *Or. diminuata* shared the same sequence and thus haplotype (Dim_1), no intraspecific genetic distance was observed between *Or. diminuata* specimens. Interspecific distances between the two analyzed species ranged from 13.0% to 14.1% (Additional file: Table S1).

## 4 Discussion

In the current study, we detected *Orthohalarachne* spp. infestations in South American sea lions by exclusively applying non-invasive methods. To the best of our knowledge, current data present the first successful development and application of non-invasive sampling techniques to detect halarachnid mite infestations in free-ranging pinnipeds. *Orthohalarachne attenuata* and *Orthohalarachne diminuata* were already reported in both free-ranging and captive South American sea lions in the past (Finnegan, 1934; Kutzer and Burtscher, 1983; Morgades et al., 2006; Gomez-Puerta and Gonzales-Viera, 2015; Seguel et al., 2018b; Ebmer et al., 2022), including co-infestations with both species (Calderón-Mayo, 2015). However, compared to other otariids like California sea lions, Northern fur seals or South American fur seals (Dahme and Popp, 1963; Kim et al., 1980; Páez, 2007; Katz et al., 2012; Gastal et al., 2016; Kuzmina et al., 2018, Kuzmina et al., 2021 Seguel et al., 2018a, 2018b; Pesapane et al., 2021), comparably few studies specifically addressed halarachnid mites in free-ranging *Otaria flavescens,* so far. Here, we present the first record of respiratory mite infestations in a group of synanthropic South American sea lions living in the city of Valdivia, Chile.

Non-invasive sampling techniques to diagnose wildlife diseases have received growing attention in recent years and new collection devices have been developed to minimize distress in sampled free-ranging animals (Schilling et al., 2022). Recently, Ebmer et al. (2019) developed a novel “telescopic lice comb”, a non-invasive device to collect echinophthiriid lice in pinnipeds, and detected *Antarctophthirus microchir* in the "urban" colony of South American sea lions. However, for various pathogens of problematic infection/infestation sites, no appropriate methods are available (Schilling et al., 2022). As such, *in vivo* diagnostics of halarachnid mites living in the respiratory tract of semiaquatic marine mammals is very challenging due to their obligatory endoparasitc lifestyle. Consequently, the vast majority of published nasopharyngeal/nasopulmonary cases in free-ranging pinnipeds and otters resulted from necropsies of stranded animals (Kim et al., 1980; Seguel et al., 2018a, 2018b; Shockling Dent et al., 2019). So far, the only non/minimally-invasive detection of halarachnid mites in pinnipeds was conducted via voluntary rhinoscopy in trained captive walruses (Fravel and Procter, 2016). For the first time, we here describe non-invasive diagnostic techniques that allow sampling of halarachnid mites to obtain important insights into patent infestations *in vivo.* Even though these techniques require time-intensive animal observations, they do not rely on capture, fixation or immobilisation of the hosts and thereby allow research in conformity with animal welfare and conservation aspects.

We here showed that sampling of freshly expectorated nasal mucus in the enviroment constitutes a chance to obtain biological material for halarachnid mite detection circumventing necropsies for diagnostics. This sampling method needs exact animal observations, but does not require direct animal contact, thereby leaving the animals rather unaffected. Since halarachnid mites occur in members of all pinniped families (Otariidae, Odobenidae, Phocidae) (Rolbiecki et al., 2018), this method could be applied in different pinniped species worldwide. For example, *Halarachne halichoeri* Allman, 1847 is a common parasite of grey seals *Halichoerus grypus* (Fabricius, 1791) (Alonso-Farré et al., 2012) and was recently reported as re-emerging in the German Wadden sea (Reckendorf et al., 2019). Considering increasing grey seal numbers and births and the close vicinity to humans on Heligoland, Germany (Abt and Engler, 2009), animal observations for respiratory symptoms and sampling of nasal secretions could constitute a possible application for the techniques described here. Further, the respiratory mite fauna of other synanthropic pinniped species, e. g. Galapagos sea lions *Zalophus wollebaeki* Sivertsen, 1953 (Denkinger et al., 2015), could be examined with these techniques. In this study*,* the vast majority of mucus samples were collected out of the environment; however, self-developed collection methods here described could constitute promising tools to sample halarachnids in other synanthropic pinnipeds. Compared to the mounted petri dishes, the square-framed clothes hanger covered with clingfilm exhibited a larger surface, thereby increasing the chance to directly trap expectorated mucus. However, direct collection of mucus samples in sterile petri dishes may also facilitate the diagnosis of other pathogens like viruses, bacteria or fungi in marine mammal screenings. For studying free-ranging pinniped populations that are not used to interactions with humans, procedures should be modified and focused on sampling of secretions out of the environment due to the shyness of these animals.

Whilst *Or. attenuata* and *Or. diminuata* adults clearly differ in their morphology, larval stages of both *Orthohalarachne* species are morphologically very similar and hard to differentiate via microscopy even though *Or. attenuata* larval body sizes are described to be larger (Domrow, 1974; Furman, 1977). However, due to the limited sample size and planned individual molecular identifications of detected mites in the current study, larvae were not mounted in embedding medium and were only analyzed on microscope slides in 80% ethanol to save material. Although idiosomal and gnathosomal lengths were measured, measurements should be interpreted as approximate values and compared with caution as the exact analysis of larval mite morpho-anatomy was impossible. Nevertheless, size differences between the two species were indeed noticeable and are in accordance with previous data (Furman, 1977). However, large *Or. diminuata* larvae, potentially prior to molting, might be misidentified as early *Or. attenuata* stages. Therefore, PCR-based methods and sequencing provide valuable tools to bridge morphological identification difficulties, especially when specimens are in poor condition.

To date, the 3′ end of the mitochondrial 16S rRNA gene (rDNA), as introduced by Black and Piesman (1994), is the most frequently used genetic marker for barcoding Acari. Yet, other genes, such as cytochrome *c* oxidase subunit 1 (COI) or ITS nuclear ribosomal DNA regions, are used for species identification or phylogenetic approaches. Particularly for *Orthohalarachne* species, sequence data are still scarce, and first 16S rDNA sequences of the two described species have been published rather recently (Pesapane et al., 2021; Ebmer et al., 2022). Therefore, we sequenced 16S rDNA as well as an additional set of sequences (18S, 28S, and ITS1) from Chilean specimens.

Despite aforementioned size variations, larval stages of *Orthohalarachne* do not exhibit marked morphological differences, thus, molecular analysis might provide a more accurate method for identification. Of seven *Orthohalarachne* specimens analyzed in this study, one specimen showed a much lower DNA yield compared to all others. This might be due to the environmental collection method, which could promote exposure of specimens to DNA degrading factors like UV light, heat or PCR inhibitors. Morphological identification should always be attempted before the application of molecular methods, as applied in our study. However, measurements need to be standardized on a high number of specimens and still allow for individual nucleic acid isolation.

All specimens were successfully identified based on the 16S marker gene. Despite a huge geographic distance, *Or. diminuata* 16S sequences of Chilean and Austrian specimens did not show any nucleotide differences (single haplotype), while 16S sequences of *Or. attenuata* from Valdivia, Chile, and California, USA, exhibited marked pairwise differences of 2.7–3.3% between the respective countries. In general, over 95% identity based on 16S is normally seen for members of the same tick (Acari: Ixodida) species (Dantas-Torres, 2018), which is also supported by the current analyses on *Or. attenuata*. Interestingly, all *Or. diminuata* sequences (Chilean and Austrian) originated from specimens infesting a single host species, namely South American sea lions, while *Or. attenuata* sequences were obtained from specimens infesting four different pinniped host species in California (Pesapane et al., 2021). Genetic diversity might be driven by infesting different, possibly sympatric, pinniped host species. It is noteworthy, that the current haplotype analysis is still rather preliminary as only a low number of available sequences could be included and, thus, should be complemented with a larger dataset and subsequently different genes in future studies. Among the genes sequenced in our study, only ITS1 showed intra-specific sequence divergence and therefore might be a good candidate for deeper genetic analyses, as shown for *Dermanyssus gallinae* (De Geer, 1778) (Brännström et al., 2008) or mesostigmatid mites of the Lealapidae family (Engelbrecht et al., 2014).

Although exact transmission modalities of halarachnid mites have not been studied yet, spreading of mites between pinnipeds can most likely occur via direct nose-to-nose contact or the scenario of sneezed mucus droplets landing in proximity of the nostrils so that hexapod larvae can crawl out of their protective mucus-coat and actively invade nasal cavities of new host individuals (Kim et al., 1980). In the current study, we exclusively found larval stages in expectorated nasal secretions, which is in accordance with the current knowledge on larval mites as epidemiologically important transmission stages (Dahme and Popp, 1963; Furman and Smith, 1973). Under experimental conditions, *Orthohalarachne* larval stages can survive outside the host for several weeks (Furman and Smith, 1973). Our observations showed massive nasal discharge of hosts, mucus expectorated over several metres by the animals and the contamination of sleeping and resting places with larvae-containing expectorations. Potentially, larvae protected in mucus-coats can linger in the environment and be attracted by CO_2_ (Furman and Smith, 1973) out of the exhaled air of other sea lion individuals, starting new infestations by crawling into their nostrils. Synanthropic South American sea lions in Valdivia were reported to spend prolonged times ashore resting in public places, to show reduced foraging behavior due to regular feedings by marketeers at the fish market and generally seem less active than their non-synanthropic conspecifics (Hermosilla et al., 2016; Ebmer et al., 2020). Furthermore, massive respiratory symptoms of “urban” sea lions, e. g. strong rhinorrhea, coughing, sneezing periods with plainly visible mucus, were observed by our group over several years; however, it is not resolved yet, if detected mite infestations can directly be associated with these observed pathologies. Nevertheless, halarachnid mites have already been presumed to facilitate secondary bacterial infections (Seguel et al., 2018a) and, recently, as potential vectors of various bacterial pathogens (Pesapane et al., 2022). We therefore call for more research in this field, especially on the impact and exact role of halarachnid mites in respiratory diseases in pinnipeds.

Aside from free-ranging animals, respiratory mites were also detected in captive pinnipeds in the past, however, mainly as accidental finding in necropsies of wild-caught animals (Eichler, 1958; Kutzer and Burtscher, 1983). Recently, our group detected a severe *Or. diminuata* infestation in a zoo-born South American sea lion held at Vienna Zoo, Austria, and confirmed the presence of an autochthonous infestation in a zoological garden (Ebmer et al., 2022). Thereby, these results essentially supported the molecular identification process of *Or. diminuata* larvae in the current study to confirm morphological identification results. Overall, the results of Ebmer et al. (2022) and the current study illustrate the importance and successful combination of *ex situ* and *in situ* research projects, that can profit from each other and lead to a better understanding of infectious diseases in wildlife. In both the wild and zoological gardens, infestations with respiratory mites in pinnipeds constitute neglected parasitoses with various unanswered research questions on occurrence, biology and potential detrimental effects that urgently need to be addressed in future studies.

## 5 Conclusions

In the current study, non-invasive diagnostic techniques were successfully developed and applied for detecting infestations with halarachnid mites in free-ranging synanthropic pinnipeds. Thereby, patent (co)-infestations in “urban” South American sea lions were detected and molecularly verified for the first time, contributing to the current knowledge on the occurrence of respiratory mites. Particularly studies on underreported mesostigmatid mite families, such as the Halarachnidae, should be accompanied with a molecular dataset that can be used for phylogenetic inference. Respiratory mites constitute neglected parasites in free-ranging and captive pinnipeds and further studies are needed to better understand their role in disease processes and their impact on pinniped and sea otter health.

## Ethics approval

Non-invasive sampling was authorized by the Chilean National Fisheries and Aquaculture Services (SERNAPESCA), the Undersecretariat for Fisheries and Aquaculture (SUBPESCA) (reference number E−2022-715) and the municipality service of the city of Valdivia, Chile. All procedures were carried out in accordance with the Chilean Animal Welfare Legislation.

## Declaration of competing interest

The authors declare that they have no competing interests.

## Appendix A Supplementary data

Fig. S1 Multiple sequence aligment of all 16S rRNA gene sequences analyzed in this study. Fig. S1

Table S1 Uncorrected pairwise 16S rRNA gene sequences of *Or. attenuata* and *Or. diminuata*. Table S1
