# Supplementary material for: Non-invasive detection of Orthohalarachne attenuata (Banks, 1910) and Orthohalarachne diminuata (Doetschman, 1944) (Acari: Halarachnidae) in free-ranging synanthropic South American sea lions Otaria flavescens (Shaw, 1800)
Source: Int J Parasitol Parasites Wildl. 2023 Jun 3;21:192–200. doi: 10.1016/j.ijppaw.2023.06.001 (PMC10422118; doi:10.1016/j.ijppaw.2023.06.001)
Supplement: Fig. S1 — Multiple sequence aligment of all 16S rRNA gene sequences analyzed in this study. [file mmc1.pdf]

**OQ376656.1 *Or. attenuata* (OC2)**	**A C A A G G C T T T G A A T G G G T T C T A A G T G A A T G A A G G A A T G G G G G A G T A C T T T**	**[50]**
**OQ376657.1 *Or. attenuata* (OC3)**	. . . .	[50]
MZ435793.1 *Or. attenuata*	. . . . A . . . . T . . . . A	[50]
MZ435795.1 *Or. attenuata*	. . . . A . . . . T . . . . A	[50]
MZ736595.1 *Or. attenuata*	. . . . A . . . . T . . . . A	[50]
MZ435797.1 *Or. attenuata*	. . . . A . . . . T . . . . A	[50]
MZ435794.1 *Or. attenuata*	. . . . A . . . . T . . . . A	[50]
MZ435798.1 *Or. attenuata*	. . . . A . . . . T . . . . A	[50]
MZ435796.1 *Or. attenuata*	. . . . A . . . . T . . . . A	[50]
**OQ376658.1 *Or. diminuata* (OC1)**	. T . . . . A . . . . A . C G . . . . G G C A T . . . . G . C . . . A .	[50]
**OQ376659.1 *Or. diminuata* (OC4)**	. T . . . . A . . . . A . C G . . . . G G C A T . . . . G . C . . . A .	[50]
**OQ592162.1 *Or. diminuata* (OC5)**	. T . . . . A . . . . A . C G . . . . G G C A T . . . . G . C . . . A .	[50]
**OQ376660.1 *Or. diminuata* (OC6)**	. T . . . . A . . . . A . C G . . . . G G C A T . . . . G . C . . . A .	[50]
**OQ376661.1 *Or. diminuata* (OC7)**	. T . . . . A . . . . A . C G . . . . G G C A T . . . . G . C . . . A .	[50]
OP087659.1 *Or. diminuata*	. T . . . . A . . . . A . C G . . . . G G C A T . . . . G . C . . . A .	[50]
OP087660.1 *Or. diminuata*	. T . . . . A . . . . A . C G . . . . G G C A T . . . . G . C . . . A .	[50]
OP087661.1 *Or. diminuata*	. T . . . . A . . . . A . C G . . . . G G C A T . . . . G . C . . . A .	[50]
OP087662.1 *Or. diminuata*	. T . . . . A . . . . A . C G . . . . G G C A T . . . . G . C . . . A .	[50]
**OQ376656.1 *Or. attenuata* (OC2)**	**C T T T A G T A A A A G A T T A G A A T T T T T C T T A A G A A T G A G G A G A T T C T T A T A A A**	**[100]**
**OQ376657.1 *Or. attenuata* (OC3)**	. . . .	[100]
MZ435793.1 *Or. attenuata*	. . . .	[100]
MZ435795.1 *Or. attenuata*	. . . .	[100]
MZ736595.1 *Or. attenuata*	. . . .	[100]
MZ435797.1 *Or. attenuata*	. . . .	[100]
MZ435794.1 *Or. attenuata*	. . . .	[100]
MZ435798.1 *Or. attenuata*	. . . .	[100]
MZ435796.1 *Or. attenuata*	. . . .	[100]
**OQ376658.1 *Or. diminuata* (OC1)**	. . . . T . . . . A . . . . . A A . A . . . T . . . . . T .	[100]
**OQ376659.1 *Or. diminuata* (OC4)**	. . . . T . . . . A . . . . . A A . A . . . T . . . . . T .	[100]
**OQ592162.1 *Or. diminuata* (OC5)**	. . . . T . . . . A . . . . . A A . A . . . T . . . . . T .	[100]
**OQ376660.1 *Or. diminuata* (OC6)**	. . . . T . . . . A . . . . . A A . A . . . T . . . . . T .	[100]
**OQ376661.1 *Or. diminuata* (OC7)**	. . . . T . . . . A . . . . . A A . A . . . T . . . . . T .	[100]
OP087659.1 *Or. diminuata*	. . . . T . . . . A . . . . . A A . A . . . T . . . . . T .	[100]
OP087660.1 *Or. diminuata*	. . . . T . . . . A . . . . . A A . A . . . T . . . . . T .	[100]
OP087661.1 *Or. diminuata*	. . . . T . . . . A . . . . . A A . A . . . T . . . . . T .	[100]
OP087662.1 *Or. diminuata*	. . . . T . . . . A . . . . . A A . A . . . T . . . . . T .	[100]

**OQ376656.1 *Or. attenuata* (OC2)**	**A T A G T G G G A C G A G A A G A C C C T A T G A A C C T T T A T A T G T A T T A T A G A A C A G A**	**[150]**
**OQ376657.1 *Or. attenuata* (OC3)**	. . . .	[150]
MZ435793.1 *Or. attenuata*	. . . . G . . G . . . . C T . . .	[150]
MZ435795.1 *Or. attenuata*	. . . . G . . G . . . . C T . . .	[150]
MZ736595.1 *Or. attenuata*	. . . . G . . G . . . . C T . . .	[150]
MZ435797.1 *Or. attenuata*	. . . . G . . G . . . . C T . . .	[150]
MZ435794.1 *Or. attenuata*	. . . . G . . G . . . . C T . . .	[150]
MZ435798.1 *Or. attenuata*	. . . . G . . . . C T . . .	[150]
MZ435796.1 *Or. attenuata*	. . . . G . . G . . . . C T . . .	[150]
**OQ376658.1 *Or. diminuata* (OC1)**	C . . . . A . . . . T . . . A . . . . T . G . A G C . A . C T G . .	[150]
**OQ376659.1 *Or. diminuata* (OC4)**	C . . . . A . . . . T . . . A . . . . T . G . A G C . A . C T G . .	[150]
**OQ592162.1 *Or. diminuata* (OC5)**	C . . . . A . . . . T . . . A . . . . T . G . A G C . A . C T G . .	[150]
**OQ376660.1 *Or. diminuata* (OC6)**	C . . . . A . . . . T . . . A . . . . T . G . A G C . A . C T G . .	[150]
**OQ376661.1 *Or. diminuata* (OC7)**	C . . . . A . . . . T . . . A . . . . T . G . A G C . A . C T G . .	[150]
OP087659.1 *Or. diminuata*	C . . . . A . . . . T . . . A . . . . T . G . A G C . A . C T G . .	[150]
OP087660.1 *Or. diminuata*	C . . . . A . . . . T . . . A . . . . T . G . A G C . A . C T G . .	[150]
OP087661.1 *Or. diminuata*	C . . . . A . . . . T . . . A . . . . T . G . A G C . A . C T G . .	[150]
OP087662.1 *Or. diminuata*	C . . . . A . . . . T . . . A . . . . T . G . A G C . A . C T G . .	[150]

**OQ376656.1 *Or. attenuata* (OC2)**	**A T C T A T A G T A T T T A T T T G G T T G G G G C G A C A G A A A A A T A G T A A G T A A C T T T**	**[200]**
**OQ376657.1 *Or. attenuata* (OC3)**	. . . .	[200]
MZ435793.1 *Or. attenuata*	. . . . A	[200]
MZ435795.1 *Or. attenuata*	. . . . A	[200]
MZ736595.1 *Or. attenuata*	. . . . A	[200]
MZ435797.1 *Or. attenuata*	. . . . . . G A	[200]
MZ435794.1 *Or. attenuata*	. . . . A	[200]
MZ435798.1 *Or. attenuata*	. . . . A	[200]
MZ435796.1 *Or. attenuata*	. . . . A	[200]
**OQ376658.1 *Or. diminuata* (OC1)**	. . T . G C T A . . A . . . . A	[200]
**OQ376659.1 *Or. diminuata* (OC4)**	. . T . G C T A . . A . . . . A	[200]
**OQ592162.1 *Or. diminuata* (OC5)**	. . T . G C T A . . A . . . . A	[200]
**OQ376660.1 *Or. diminuata* (OC6)**	. . T . G C T A . . A . . . . A	[200]
**OQ376661.1 *Or. diminuata* (OC7)**	. . T . G C T A . . A . . . . A	[200]
OP087659.1 *Or. diminuata*	. . T . G C T A . . A . . . . A	[200]
OP087660.1 *Or. diminuata*	. . T . G C T A . . A . . . . A	[200]
OP087661.1 *Or. diminuata*	. . T . G C T A . . A . . . . A	[200]
OP087662.1 *Or. diminuata*	. . T . G C T A . . A . . . . A	[200]

Sequence	Alignment	Position
**OQ376656.1 *Or. attenuata* (OC2)**	**A T T A T G A A A G T T T - A T T A A A A A T T G A T G G A T A A G G T A C T C T A G G G A T A A C**	**[250]**
**OQ376657.1 *Or. attenuata* (OC3)**	. . . . -	[250]
MZ435793.1 *Or. attenuata*	. . . . C . . . . T . A	[250]
MZ435795.1 *Or. attenuata*	. . . . C . . . . T . A	[250]
MZ736595.1 *Or. attenuata*	. . . . C . . . . T . A	[250]
MZ435797.1 *Or. attenuata*	. . . . T . A	[250]
MZ435794.1 *Or. attenuata*	. . . . C . . . . T . A	[250]
MZ435798.1 *Or. attenuata*	. . . . T . A	[250]
MZ435796.1 *Or. attenuata*	. . . . C . . . . T . A	[250]
**OQ376658.1 *Or. diminuata* (OC1)**	. . . . - . . . . C . . . . A . . . A	[250]
**OQ376659.1 *Or. diminuata* (OC4)**	. . . . - . . . . C . . . . A . . . A	[250]
**OQ592162.1 *Or. diminuata* (OC5)**	. . . . - . . . . C . . . . A . . . A	[250]
**OQ376660.1 *Or. diminuata* (OC6)**	. . . . - . . . . C . . . . A . . . A	[250]
**OQ376661.1 *Or. diminuata* (OC7)**	. . . . - . . . . C . . . . A . . . A	[250]
OP087659.1 *Or. diminuata*	. . . . - . . . . C . . . . A . . . A	[250]
OP087660.1 *Or. diminuata*	. . . . - . . . . C . . . . A . . . A	[250]
OP087661.1 *Or. diminuata*	. . . . - . . . . C . . . . A . . . A	[250]
OP087662.1 *Or. diminuata*	. . . . - . . . . C . . . . A . . . A	[250]

Sequence	Alignment	Position
**OQ376656.1 *Or. attenuata* (OC2)**	**A G C G T G A T T A T T T T G G A A A G G C C A T A T T G A T A A A A T A G T T T G C G A C C T C G**	**[300]**
**OQ376657.1 *Or. attenuata* (OC3)**	. . . .	[300]
MZ435793.1 *Or. attenuata*	. . . . T	[300]
MZ435795.1 *Or. attenuata*	. . . . T	[300]
MZ736595.1 *Or. attenuata*	. . . . T	[300]
MZ435797.1 *Or. attenuata*	. . . . T	[300]
MZ435794.1 *Or. attenuata*	. . . . T	[300]
MZ435798.1 *Or. attenuata*	. . . . T	[300]
MZ435796.1 *Or. attenuata*	. . . . T	[300]
**OQ376658.1 *Or. diminuata* (OC1)**	. . . . . A . . . . A	[300]
**OQ376659.1 *Or. diminuata* (OC4)**	. . . . . A . . . . A	[300]
**OQ592162.1 *Or. diminuata* (OC5)**	. . . . . A . . . . A	[300]
**OQ376660.1 *Or. diminuata* (OC6)**	. . . . . A . . . . A	[300]
**OQ376661.1 *Or. diminuata* (OC7)**	. . . . . A . . . . A	[300]
OP087659.1 *Or. diminuata*	. . . . . A . . . . A	[300]
OP087660.1 *Or. diminuata*	. . . . . A . . . . A	[300]
OP087661.1 *Or. diminuata*	. . . . . A . . . . A	[300]
OP087662.1 *Or. diminuata*	. . . . . A . . . . A	[300]

**OQ376656.1 *Or. attenuata* (OC2)**	**A T G T T G A A T T A G T G C A C C T C T T T A G C C G C A G T T G G T T A G G A A G G C A G A T T**	**[350]**
**OQ376657.1 *Or. attenuata* (OC3)**	. . . .	[350]
MZ435793.1 *Or. attenuata*	. . . .	[350]
MZ435795.1 *Or. attenuata*	. . . .	[350]
MZ736595.1 *Or. attenuata*	. . . .	[350]
MZ435797.1 *Or. attenuata*	. . . .	[350]
MZ435794.1 *Or. attenuata*	. . . .	[350]
MZ435798.1 *Or. attenuata*	. . . .	[350]
MZ435796.1 *Or. attenuata*	. . . .	[350]
**OQ376658.1 *Or. diminuata* (OC1)**	. . . . G . . . . T C . . . . C A . . . . . . A	[350]
**OQ376659.1 *Or. diminuata* (OC4)**	. . . . G . . . . T C . . . . C A . . . . . . A	[350]
**OQ592162.1 *Or. diminuata* (OC5)**	. . . . G . . . . T C . . . . C A . . . . . . A	[350]
**OQ376660.1 *Or. diminuata* (OC6)**	. . . . G . . . . T C . . . . C A . . . . . . A	[350]
**OQ376661.1 *Or. diminuata* (OC7)**	. . . . G . . . . T C . . . . C A . . . . . . A	[350]
OP087659.1 *Or. diminuata*	. . . . G . . . . T C . . . . C A . . . . . . A	[350]
OP087660.1 *Or. diminuata*	. . . . G . . . . T C . . . . C A . . . . . . A	[350]
OP087661.1 *Or. diminuata*	. . . . G . . . . T C . . . . C A . . . . . . A	[350]
OP087662.1 *Or. diminuata*	. . . . G . . . . T C . . . . C A . . . . . . A	[350]

**OQ376656.1 *Or. attenuata* (OC2)**	**G T T C A T C T G T T G A A T C A C T**	**[369]**
**OQ376657.1 *Or. attenuata* (OC3)**	. . . .	[369]
MZ435793.1 *Or. attenuata*	. . . . A	[369]
MZ435795.1 *Or. attenuata*	. . . . A	[369]
MZ736595.1 *Or. attenuata*	. . . . A	[369]
MZ435797.1 *Or. attenuata*	. . . . A	[369]
MZ435794.1 *Or. attenuata*	. . . . A	[369]
MZ435798.1 *Or. attenuata*	. . . . A	[369]
MZ435796.1 *Or. attenuata*	. . . . A	[369]
**OQ376658.1 *Or. diminuata* (OC1)**	. . . . A	[369]
**OQ376659.1 *Or. diminuata* (OC4)**	. . . . A	[369]
**OQ592162.1 *Or. diminuata* (OC5)**	. . . . A	[369]
**OQ376660.1 *Or. diminuata* (OC6)**	. . . . A	[369]
**OQ376661.1 *Or. diminuata* (OC7)**	. . . . A	[369]
OP087659.1 *Or. diminuata*	. . . . A	[369]
OP087660.1 *Or. diminuata*	. . . . A	[369]
OP087661.1 *Or. diminuata*	. . . . A	[369]
OP087662.1 *Or. diminuata*	. . . . A	[369]
